# Chemokine Receptor and Ligand Upregulation in the Diaphragm during Endotoxemia and Pseudomonas Lung Infection

**DOI:** 10.1155/2009/860565

**Published:** 2009-04-29

**Authors:** Alexandre Demoule, Maziar Divangahi, Linda Yahiaoui, Gawiyou Danialou, Dusanka Gvozdic, Basil J. Petrof

**Affiliations:** ^1^Meakins-Christie Laboratories, McGill University, Montreal, QC, Canada H2X 2P2; ^2^UPRES EA2397 Université, Pierre et Marie Curie, 75005 Paris, France; ^3^Respiratory Division, McGill University Health Centre, Royal Victoria Hospital, 687 Pine Avenue West, Montreal, QC, Canada H3A 1A1

## Abstract

Sepsis-induced diaphragmatic inflammation has been associated with respiratory failure, but the role of chemokines in this process has not been evaluated. Here we sought to study the local expression and molecular regulation of the chemokines, regulated upon activation normal T cell expressed and secreted (RANTES) and macrophage inflammatory protein (MIP)-1*α*, in the murine diaphragm during sepsis. Constitutive expression levels of RANTES and MIP-1*α*, as well as their receptors, CCR1 and CCR5, were significantly higher in diaphragm than limb muscle. Sepsis was induced by acute lipopolysaccharide (LPS) delivery or subacutely by intratracheal administration of live *Pseudomonas aeruginosa* bacteria. Both sepsis models triggered a marked upregulation of RANTES and MIP-1*α* in the diaphragm. In vitro, stimulation of diaphragmatic muscle cells with LPS also led to RANTES upregulation. Inhibition of the NF-kB pathway using pharmacologic or dominant negative genetic approaches blocked the LPS-induced RANTES upregulation, while free radical scavengers had no effect. We conclude that sepsis leads to greatly increased expression of RANTES, MIP-1*α* and their cognate receptors in the diaphragm. Manipulation of the NF-kB pathway and other regulators of chemokine expression in the diaphragm could represent a novel method for mitigating the skeletal muscle inflammatory response associated with sepsis-induced diaphragmatic dysfunction.

## 1. Introduction

Diaphragmatic dysfunction likely plays an important role in sepsis-related respiratory failure, a condition which occurs in a large proportion of patients admitted to the ICU for severe sepsis [1]. Sepsis-induced diaphragmatic dysfunction is a pleiomorphic phenomenon manifested by decreased force-generating capacity of the muscle, an increased susceptibility to fatigue, and the presence of muscle fiber injury [2–7]. Inflammatory mechanisms have been strongly implicated, with oxygen free radicals [8–10], nitric oxide or its derivatives [4, 5], and TNF-*α* [11, 12] being amongst the major mediators identified thus far. 

The potential involvement of chemokines in this process has not been examined to date. Chemokines are low-molecular-weight cytokines that play a key role in orchestrating leukocyte trafficking to infected or diseased tissues. The chemokines are subdivided into four main families (CC, CXC, CX3C, and C) based upon their structure [13]. Increased expression levels of several chemokine ligands and their cognate receptors, particularly of the CC family, have been found in muscle biopsies obtained from animal models and human patients suffering from muscular dystrophy or inflammatory myopathies [14–16]. In this regard, we have recently reported a marked upregulation of CCR1 and two of its major ligands, RANTES (CCL5) and MIP-1*α* (CCL3), in the diaphragms of muscular dystrophy (mdx) mice and demonstrated a relationship with the degree of muscle infiltration by mononuclear inflammatory cells [16]. 

In addition to their classical role as leukocyte chemoattractants, the chemokines influence a diverse repertoire of normal and pathophysiologic processes, including myoblast behavior in skeletal muscle repair [17–19] and tissue responses to oxidative stress [20]. RANTES and MIP-1*α* can also act together with IFN-*γ* to synergistically upregulate TNF-*α* [21], the most prototypical cytokine implicated in sepsis-induced diaphragmatic dysfunction. Furthermore, it has recently been demonstrated that the CCR1-RANTES axis is a key modulator of the host immune response to bacterial peritonitis [22] as well as exogenously administered LPS [23].

In the present study, our primary objective was to determine whether RANTES and MIP-1*α*, as well as their major receptors, are upregulated within the diaphragm after the induction of sepsis in vivo. To examine this question, we employed two different models of sepsis in vivo, an acute model of systemic LPS administration and a more “clinically realistic” model of sustained sepsis caused by Pseudomonas lung infection. We further dissected the cellular mechanisms underlying chemokine upregulation in the septic diaphragm, by examining the responses of primary diaphragm muscle cells to LPS stimulation in vitro. More specifically, we sought to ascertain whether these responses could be influenced by inhibiting either free radicals or the NF-*κ*B transcription factor pathway, since both have been implicated in sepsis-associated respiratory muscle dysfunction.

## 2. Materials and Methods

### 2.1. Animal Models of Sepsis

All animal procedures were approved by the institutional animal care committee. Male C57BL/6 mice (Charles River, Quebec, Canada) aged 8–10 weeks (23–26 g) were studied. Anesthesia was achieved with a mixture of ketamine (Fort Dodge Animal Health, Fort Dodge, IA) and xylazine (Bayer, Shawnee Mission, KS). Two experimental models of infection were used: acute endotoxemia and Pseudomonas aeruginosa lung infection. To produce endotoxemia, animals received an intraperitoneal injection of LPS (25 mg/kg of serotype O55:B5; Sigma Chemical Co., St Louis, MO) diluted in saline. In previous studies, such a dose has been shown to produce a significant impairment of diaphragmatic force generation [24]. A sham-treated control group underwent the same procedure with an equivalent volume of saline alone, and all mice were euthanized at either 6 or 24 hours after injection. 

The model of chronic pulmonary infection with P. aeruginosa was performed as originally reported by Starke et al. [25], with minor modifications as we have previously described in detail [7]. The bacteria were entrapped within agar beads (100–150 *μ*m), and the number of viable bacteria was measured by homogenizing the beads and then plating 10-fold serial dilutions on trypticase soy agar plates. Sterile agar beads were produced in the same manner but with omission of bacteria and were confirmed to be free of colony-forming units (CFUs). The trachea of anesthetized mice was intubated to deliver either bacteria-containing or sterile agar beads to mouse lungs, and the mice were killed at either 2 or 7 days later. Two different doses of inoculating bacteria (2 × 105 and 1 × 106 cfu) were studied at 2 days after infection, whereas only the lower inoculating dose was used for the 7-day time point due to unacceptable signs of ill health at the higher dose in the 7-day group. The higher dose has been shown to produce a significant impairment of diaphragm force generation 2 days after infection, whereas the lower dose has been shown to produce similar effects 7 days after infection [7].

### 2.2. Primary Muscle Cell Cultures

Primary diaphragm and tibialis anterior (TA) muscle cell cultures were established as previously described in detail [16], using single living muscle fibers to isolate adult myoblasts. Briefly, excised diaphragm muscle strips were digested (0.2% collagenase), followed by trituration to liberate individual fibers. The fibers were transferred into Matrigel (Becton Dickinson, Franklin Lakes, NJ)-coated (1 mg/mL in DMEM) 6-well plates. Cultures were maintained in DMEM with 10% horse serum and 0.5% chick embryo extract (MP Biomedical, Aurora, OH) for 4 days, during which myoblasts attached to the substratum. Diaphragmatic myoblasts were then expanded in growth medium (20% fetal bovine serum, 10% horse serum, 1% chick embryo extract in DMEM) until attaining approximately 75% confluence. At this point, the cultures were placed in differentiation medium (2% fetal bovine serum, 10% horse serum, 0.5% chick embryo extract in DMEM) in order to induce myoblast fusion into differentiated myotubes. All experiments were performed on the fifth day in differentiation medium. 

For LPS stimulation, the cells were washed with DMEM and then stimulated during 4 hours with E. Coli LPS (1 *μ*g/mL). To study the involvement of NF-*κ*B, myotubes were treated one hour before LPS exposure with pyrrolidine dithiocarbamate (PDTC; at 100 *μ*M) (Fisher Scientific, Nepean, ON), a compound which inhibits the ubiquitin E3 ligase function required for NF-*κ*B activation [26, 27]. To study the potential role of free radicals, myotubes were treated with catalase (at 2000 U/mL) or N-acetylcysteine (NAC; at 10 mM) (Fisher Scientific, Nepean, ON), initiated one hour before LPS exposure [28].

### 2.3. Muscle Cell Transfections

Because the transfection efficiency of primary muscle cell cultures is very low [29], a murine myoblast cell line (C2C12) was employed for transfection experiments. C2C12 cells (5 × 105) were seeded onto 60 mm plates, incubated overnight in growth medium (DMEM-10% FBS), and transfected the following day at approximately 60% confluence. To achieve transfection, Lipofectamine 2000TM (Invitrogen, Carlbad, CA) and 8 *μ*g of plasmid DNA were combined according to the manufacturer's protocol. At 48 hours posttransfection, the cells were trypsinized, replated in 6-well plates and allowed to attach overnight. The next day the medium was switched to DMEM-2% HS to induce differentiation, and LPS stimulation experiments were performed on differentiated myotubes 5 days later, as described earlier for primary cells. Cells transfected with pEGFP-C1 (BD Bioscience, Clontech, San Diego, CA), expressing green fluorescent protein (GFP), served as transfection controls.

To evaluate transcriptional activity from the RANTES promoter, muscle cells were transfected with a firefly luciferase expression plasmid under the control of the RANTES promoter (gift from Moriuchi et al. [30]). The cells were exposed to LPS for 4 hours, then lysed and assayed for luciferase activity. To assess the involvement of NF-*κ*B in chemokine gene regulation, dominant negative mutant forms of the I*κ*B kinases (gift from Bhakar et al. [31]), IKK*α*, and IKK*β* were employed to inhibit NF-*κ*B activation. 

### 2.4. Ribonuclease Protection Assays

The mRNA expression levels of CCR1 and CCR5 as well as their two major ligands, RANTES and MIP-1*α*, were assessed in RNA extracted from tissues or muscle cell cultures using Trizol (Invitrogen, Carlsbad, CA). The labelled riboprobes were hybridized with total RNA overnight (20 micrograms from muscle tissues and 5 micrograms from muscle cell cultures) and then processed using the manufacturer's protocol (BD Bioscience, Pharmingen, San Diego, CA). Protected mRNAs were separated using a 5% acrylamide gel (19:1 acrylamide:bisacrylamide). Gels were transferred to filter paper and dried under vacuum using a gel drier. Dried gels were exposed to scientific X-ray imaging film (Kodak, Rochester, NY), and the bands were quantified using an image analysis system (FluorChem 8000, Alpha Innotech Corp, San Leandro, CA). All signals were normalized to L32 as a loading control and expressed in absolute arbitrary units. 

### 2.5. Statistical Analyses

Values are expressed as means ± SE. For both in vivo and in vitro studies, 6 independent replicate analyses were performed for each experimental condition and time point, unless otherwise noted. For the same experimental condition, diaphragm and TA were compared using the Wilcoxon sign rank test. For the same type of muscle, LPS and saline groups were compared using the Mann-Whitney Rank Sum test. Comparisons between three or more groups were performed using the Kruskall-Wallis test. When significant, post hoc multiple comparisons were archived using the Student-Newman-Keuls method. The analysis was performed using SigmaStat software (SPSS, Chicago, IL), and statistical significance was set at *P* < .05.

## 3. Results

### 3.1. Endotoxemia Upregulates Expression of CCR1 and Its Ligands, RANTES and MIP-1*α*, within the Diaphragm In Vivo

**Figure 1:**
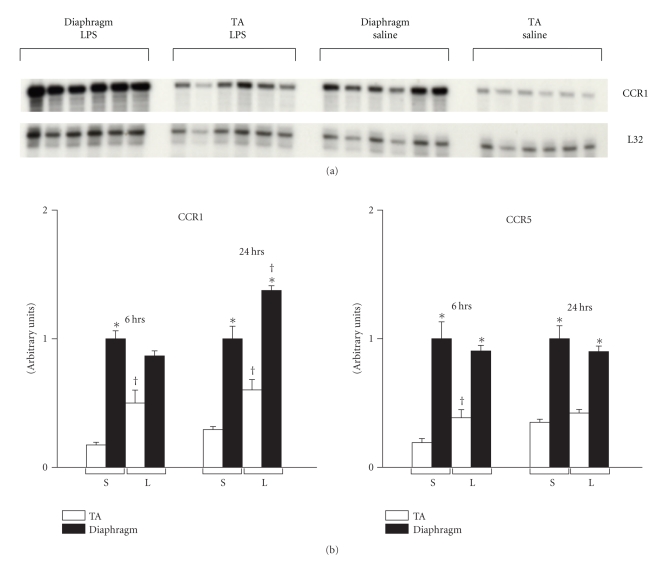
CC-class chemokine receptor gene expression in the diaphragm and limb muscle at baseline and during endotoxemia in vivo. (a) Representative autoradiograph of ribonuclease protection assay data, showing CCR1 gene expression in the mouse diaphragm and tibialis anterior (TA) muscles at 24 hours following injection of either LPS or saline. (b) Quantification of CCR1 and CCR5 mRNA levels at 6 and 24 hours after LPS administration. Note that the samples for each time point were loaded onto separate gels, such that the absolute arbitrary units depicted are valid for quantitative comparisons within but not between time points. All data are group means ± SE, *n* = 6 mice per group. Data are normalized to the L32 housekeeping gene. *P < .05 for comparisons between diaphragm and TA within the same condition (LPS or sham control); ^†^ *P* < .05 for comparisons between LPS- and sham-control- (saline-) treated mice within the same type of muscle (diaphragm or TA).

To ascertain the effects of endotoxemia on CCR1 and CCR5 expression in the diaphragm and TA muscles, total RNA was extracted at 6 or 24 hours after LPS administration and analyzed by ribonuclease protection assays. A representative autoradiograph showing CCR1 expression levels at 24 hours after LPS delivery, as well as group mean data (expressed in arbitrary units) for both time points, are provided in Figure 1. It should be noted that for these experiments, all of the RNA samples evaluated for a specific time point (*n* = 24) were loaded onto the same gel in order to ensure that the X-ray exposure conditions were identical. This allowed for valid quantitative comparisons at a given time point but not necessarily between different time points, and thus only the former was subjected to statistical analysis. As can be seen, CCR1 and CCR5 were both expressed at a higher constitutive level in the diaphragm than in the TA limb muscle under control (saline) conditions. However, only CCR1 showed upregulation in the diaphragm during sepsis, and this was observed at the 24-hour time point. Interestingly, both CCR1 and CCR5 were significantly upregulated in the TA muscle at 6 hours after LPS administration. 

**Figure 2:**
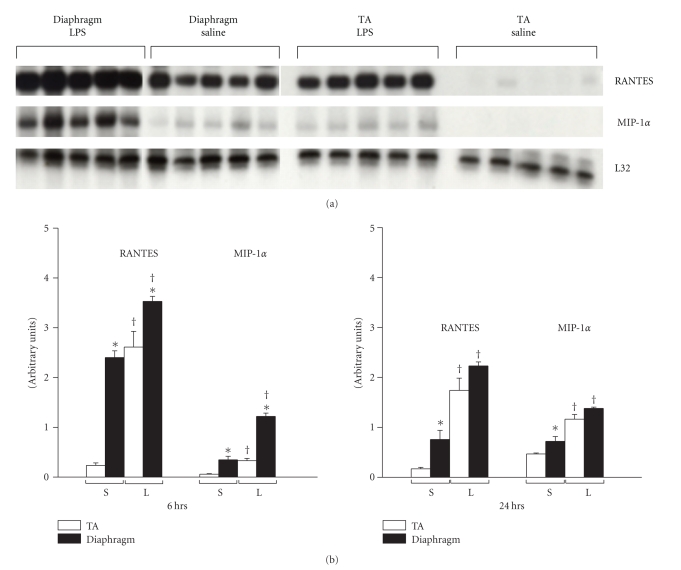
Effects of endotoxemia on RANTES and MIP-1*α* gene expression in the diaphragm and limb muscle. (a) Ribonuclease protection assay demonstrating RANTES and MIP-1*α* gene expression in the diaphragm and TA at 24 hours following LPS or saline administration. (b) Quantification of RANTES and MIP-1*α* mRNA levels at different time points after LPS delivery. All data are group means ± SE, *n* = 6 mice per group. Data are normalized to the L32 housekeeping gene. *P < .05 for comparisons between diaphragm and TA within the same condition (LPS or sham control); ^†^ *P* < .05 for comparisons between LPS- and sham control (saline)-treated mice within the same type of muscle (diaphragm or TA).

We next sought to evaluate whether the observed upregulation of CCRs in the septic diaphragm and TA is associated with altered expression of their cognate ligands, RANTES and MIP-1*α*, at the same time points (6 and 24 hours) after LPS delivery in vivo. Figure 2 shows that the overall patterns of RANTES and MIP-1*α* gene expression responses were similar. Hence for both chemokines, increased mRNA expression was induced by LPS injection in the diaphragm at 6 hours, and levels remained elevated over control values at 24 hours. In addition, under control conditions, the baseline expression levels of both RANTES and MIP-1*α* were significantly higher in the diaphragm than in the TA limb muscle. Similarly, the absolute magnitude of mRNA expression for both chemokines after LPS administration was generally greater in the diaphragm than in the limb muscle.

### 3.2. RANTES and MIP-1*α* are Upregulated in the Diaphragm during Sustained Pseudomonas Lung Infection

**Figure 3:**
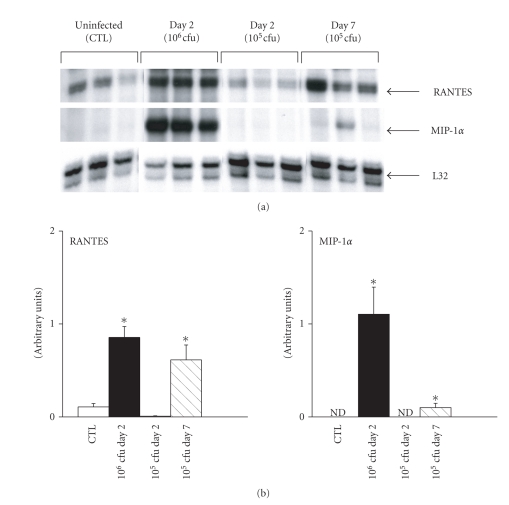
Bacterial dose- and time-dependence of chemokine gene expression in the diaphragm during Pseudomonas lung infection. (a) Representative ribonuclease protection assay data indicating RANTES and MIP-1*α* mRNA levels in the diaphragm at two separate time points (Day 2 and Day 7) following intratracheal instillation of Pseudomonas aeruginosa bacteria into the lungs at different doses (5 × 105 or 1 × 106 cfu). (b) Quantification of RANTES and MIP-1*α* gene expression under the above conditions. Note that only the higher inoculating dose of bacteria led to measurable chemokine upregulation at Day 2, while the lower dose was also able to induce a significant increase in RANTES gene expression by Day 7 postinfection. All data are group means ± SE, *n* = 6 mice per group. Data are normalized to the L32 housekeeping gene. *P < .05 for comparisons to the sham control (CTL) condition. ND = signal not detectable.

To determine if similar patterns of chemokine upregulation within the diaphragm are observed in another septic situation relevant to the ICU setting, namely, gram-negative bacterial pneumonia, a live infection with Pseudomonas aeruginosa was introduced into the lungs. Two different inoculating doses were studied at Day 2 postinfection, whereas only the lower inoculating dose was used for Day 7 due to excessive mortality at this time point with the higher dose. Figure 3 shows that both RANTES and MIP-1*α* mRNA expression were upregulated at Day 2 postinfection with the higher inoculating dose (10^6^ cfu) only. In contrast, the lower inoculating dose (10^5^ cfu) also triggered an increased expression of the two chemokines at Day 7 postinfection, and this was particularly striking in the case of RANTES. Interestingly, we did not observe any upregulation of either MIP-1*α* and RANTES within limb muscles under the same conditions (data not shown), and the levels of chemokine upregulation in the diaphragm at both time points after Pseudomonas infection were greater (relative to control) than following acute LPS administration.

### 3.3. Cellular Mechanisms of Chemokine Gene Upregulation in the Septic Diaphragm

**Figure 4:**
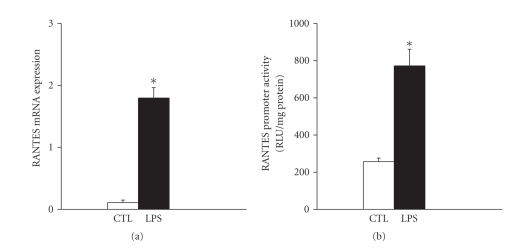
LPS is able to activate upregulation of the RANTES gene in cultured muscle cells. (a) RANTES mRNA levels in primary diaphragmatic myotubes under basal conditions (CTL) and in response to a 4-hour period of exposure to LPS (1 *μ*g/mL). All data are normalized to the L32 housekeeping gene. *P < .05 for comparisons to the control (CTL) condition. (b) RANTES promoter activation as reflected by luciferase activity in C2C12 muscle cells previously transfected with a RANTES promoter-luciferase reporter gene plasmid. Myotubes were again analyzed under basal conditions (CTL) and following a 4-hour period of stimulation with LPS (1 *μ*g/mL). *P < .05 for comparisons to the control (CTL) condition.

Having shown that both RANTES and MIP-1*α* are upregulated in the diaphragm by two different sepsis models in vivo, we next wished to establish whether stimulation by LPS has direct effects on the expression of these chemokines by diaphragm muscle cells in vitro. Ribonuclease protection assays were performed to quantify RANTES and MIP-1*α* expression levels in primary diaphragm muscle cell cultures (differentiated myotubes) at 4 hours poststimulation with LPS. In the absence of LPS stimulation, RANTES expression by diaphragmatic myotubes was observed, but only at a very low level, whereas MIP-1*α* was not detectable. However, exposure of the cultured diaphragmatic myotubes to LPS markedly upregulated RANTES (see Figure 4(a)) but had no measurable effect on MIP-1*α* expression. 

To further explore the mechanism of RANTES upregulation by muscle cells after LPS stimulation, we transfected C2C12 cells (skeletal muscle cell line) with a plasmid containing the RANTES promoter linked to a luciferase reporter gene, and the transfected cells were then once again stimulated with LPS at the myotube stage. As shown in Figure 4(b), there was a significant increase in luciferase expression in the LPS-stimulated group in comparison to nonstimulated control cultures transfected with the same plasmid (transfection efficiency of approximately 50% in both groups). These data suggest that LPS-induced upregulation of RANTES expression in muscle cells involves an increased level of transcription from the RANTES promoter.

**Figure 5:**
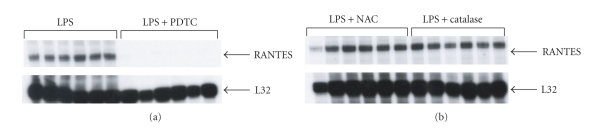
LPS-induced activation of RANTES is abrogated by pharmacological inhibition of NF-*κ*B but not reactive oxygen species. (a) The NF-*κ*B inhibitor, PDTC, dramatically suppressed RANTES gene expression in primary diaphragmatic myotubes exposed to LPS. (b) In contrast, two classical antioxidant compounds, N-acetylcysteine (NAC) and catalase, had no measurable effect on RANTES gene expression by diaphragmatic myotubes exposed to LPS under the same conditions. The primary myotube cultures were pretreated with PDTC or the antioxidant compounds for one hour prior to initiating LPS stimulation for 4 hours (1 *μ*g/mL).

Because the RANTES promoter is a target for the transcription factor NF-*κ*B, we also examined the effects of an NF-*κ*B inhibitor (PDTC) on RANTES gene expression in primary cultured diaphragmatic myotubes stimulated with LPS. Figure 5(a) demonstrates that PDTC dramatically downregulated the expression level of RANTES. These findings suggest that NF-*κ*B plays an important role in LPS-induced RANTES expression by diaphragmatic myotubes. On the other hand, NAC and catalase, two potent antioxidant compounds, had no measurable effects on RANTES expression (Figure 5(b)), suggesting that the free radical generation which is known to occur in septic diaphragm muscle [3, 9] is not a major factor in LPS-induced RANTES upregulation. 

**Figure 6:**
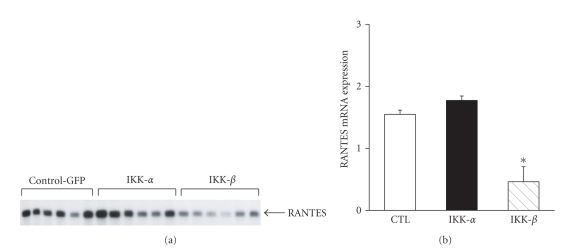
Effects of dominant negative inhibition of the NF-*κ*B pathway on LPS-induced RANTES gene expression. (a) Ribonuclease protection assay showing the effects of transfecting expression plasmids encoding dominant negative constructs for IKK*α* and IKK*β*, on RANTES expression by C2C12 myotubes exposed to LPS. Control cells were transfected in an identical fashion with a control plasmid (CTL-GFP) to determine transfection efficiency. (b) Quantification of RANTES mRNA levels in LPS-stimulated myotubes previously transfected with IKK*α* or IKK*β* dominant negative mutant constructs. All data are normalized to the L32 housekeeping gene. *P < .05 for comparisons to the CTL-GFP group.

Finally, the role of NF-*κ*B in the LPS-induced RANTES gene upregulation within differentiated myotubes was further studied using a strategy of dominant negative inhibition. Two different dominant negative constructs were transfected into C2C12 cells in order to inhibit IKK*α* and IKK*β*, which are involved in the alternate and classical NF-*κ*B pathways, respectively [32]. Figure 6 illustrates and confirms the role of the classical NF-*κ*B pathway in LPS-induced RANTES upregulation by muscle cells. Hence when C2C12 muscle cells were transfected with the dominant negative IKK*β* construct prior to LPS stimulation at the myotube stage, there was a significant inhibition of subsequent LPS-induced RANTES expression, whereas IKK*α* inhibition had no significant effect.

## 4. Discussion

Chemokines are responsible for modulating multiple aspects of the host inflammatory response against infection, such as leukocyte adhesion and migration, inflammatory cell activation, and the balance between type I and type II cytokine production [21, 33]. Serum levels of both RANTES and MIP-1*α* are elevated in septic patients [34], and modulating the biological activity of these chemokines has been shown to alter the clinical course of sepsis in animal models [22, 35]. Two major receptors targeted by RANTES and MIP-1*α* in vivo are CCR1 and CCR5, which are both expressed by a broad spectrum of leukocytes. The authors [36] and Sell et al. [37] have recently demonstrated both expression and function of these receptors in isolated skeletal muscle cells. 

To our knowledge, this is the first study to demonstrate an upregulation of CC class chemokine receptors and their ligands within septic skeletal muscles. The major findings of our study can be summarized as follows: (1) diaphragmatic CCR1 gene expression is upregulated in response to acute endotoxemia; (2) both acute endotoxemia and Pseudomonas lung infection lead to increased expression of the CCR1 ligands, RANTES and MIP-1*α*, and this occurs to a significantly greater extent within the diaphragm as compared to limb muscle; (3) LPS-induced upregulation of RANTES expression by diaphragmatic muscle cells is transcriptionally regulated and dependent upon the NF-*κ*B transcription factor pathway, whereas oxygen free radicals do not appear to be involved in this process. 

In the present investigation, two different experimental models of sepsis were employed. Both of these models have previously been shown to be associated with sepsis-induced upregulation of a number of proinflammatory cytokines (see Table 1), along with diaphragmatic dysfunction. The acute administration of LPS has the advantage of being a well-established and easily quantifiable septic stimulus, whereas the Pseudomonas lung infection model may be considered closer to most clinical situations, since it encompasses additional virulence factors within the bacteria besides endotoxin. Interestingly, in both models, the sepsis-induced chemokine upregulation was more marked in the diaphragm than in limb muscles. This pattern of preferential diaphragmatic involvement mirrors the muscle functional impairment and injury observed in several previous studies [4, 5, 7]. Moreover, the fact that the diaphragm showed greater constitutive as well as induced levels of chemokine receptor/ligand upregulation is consistent with an emerging concept that the diaphragm may be in certain respects immunologically “primed” to respond in a more vigorous fashion to inflammatory stimuli, perhaps to aid in its role as a physical barrier to the passage of infectious agents between the peritoneal and pleural cavities [38]. We have recently reported that RANTES and MIP-1*α* are also upregulated to a greater extent in the inflammatory milieu of the muscular dystrophic diaphragm than in limb muscles from the same animals [16]. In the present study, RANTES also demonstrated more sustained upregulation than MIP-1*α* in the chronic inflammatory situation associated with Pseudomonas lung infection. This is consistent with previous data showing an important role for RANTES in sustaining chronic inflammation [39, 40]. 

Stimulation of cultured primary diaphragmatic (as well as C2C12) muscle cells with LPS triggered an upregulation of RANTES expression. Interestingly, we did not detect an upregulation of MIP-1*α* within muscle cells following exposure to LPS in vitro, suggesting that sepsis-induced MIP-1*α* expression by the diaphragm in vivo may originate from nonmuscle cell types within the whole muscle. We have previously reported that primary diaphragmatic myotubes also demonstrate significant upregulation of CCR1 but not CCR5 during proinflammatory cytokine stimulation in vitro [16], which is in keeping with the greater upregulation of CCR1 during endotoxemia shown in the present study. These observations reinforce the idea that skeletal muscle fibers have the ability to behave as “immune cells” and thus actively participate in the response to various inflammatory stimuli. For example, in cultured human muscle cells, proinflammatory cytokines induce the expression of class I and class II major histocompatibility molecules [41]. Indeed, skeletal muscle cells have the ability to express a large repertoire of immunologically relevant molecules [38, 41–43]. 

To begin to dissect the molecular events underlying RANTES upregulation within septic diaphragmatic muscle cells, we first determined that the RANTES promoter is responsive to LPS in skeletal muscle cells, thus pointing to a transcriptional mechanism. We then focused on examining the role of NF-*κ*B, a transcription factor that has been shown to activate a large number of proinflammatory genes in various cell types, including RANTES [30]. In addition, inhibition of the IKK*β*-dependent NF-*κ*B has recently been shown to ameliorate muscle pathology in the setting of muscular dystrophy [44] and after experimentally induced muscle injury [45]. In our study, two distinct methods were used to evaluate the involvement of NF-*κ*B: pharmacological inhibition with PDTC and a dominant negative transfection approach. This combination of complementary methods allowed us to inhibit two critical protein complexes which are required for the phosphorylation and degradation of I*κ*B: the IKK and E3 ubiquitin ligase complexes, respectively. The sequential actions of these two complexes permit the liberation and subsequent translocation of NF-*κ*B into the nucleus in order to activate proinflammatory gene transcription [32]. In keeping with our hypothesis, both pharmacological and dominant negative molecular inhibitions of the IKK*β*-dependent classical NF-*κ*B pathway were effective in suppressing LPS-induced upregulation of RANTES. On the other hand, inhibition of the IKK*α*-dependent alternative NF-*κ*B pathway, and blockade of free radicals with the antioxidants NAC or catalase, did not have any effect on LPS-induced RANTES gene expression under the same experimental conditions, strongly suggesting that these pathways were not involved. 

What are the precise functional roles played by CC-class chemokine upregulation within the septic diaphragm muscle? The results of our study do not allow this question to be definitively addressed, since this would require a model in which muscle tissue-specific knockout of the receptor-ligand interaction could be achieved in vivo. However, there are a number of plausible scenarios. The most intuitively obvious role for CC chemokines in this setting would be to stimulate the recruitment of inflammatory cells, as has been proposed in several other diseases involving skeletal muscle [14–16]. For example, in the mdx mouse, a murine model of Duchenne muscular dystrophy, the diaphragm exhibits a pronounced infiltration by macrophages and lymphocyctes, which parallels a marked overexpression of RANTES and MIP-1*α* [16]. This is consistent with the inflammatory cell types known to be attracted by these chemokines [33]. 

In animal models of sepsis, infiltration of the diaphragm by inflammatory cells has been observed in some but not all studies. Hence in models of acute endotoxemia and bacterial peritonitis induced by cecal ligation and perforation, macrophage infiltration has been reported in the diaphragm [4, 5]. The presence of inflammatory cells in the diaphragm was associated with contractile impairment and muscle fiber injury, both of which were partially prevented by inhibition of nitric oxide synthase [4, 5]. Along these same lines, Supinski et al. [10] reported neutrophilic infiltration of the diaphragm after systemic delivery of LPS, along with an improvement in diaphragmatic contractility when the superoxide-generating NADPH complex was inhibited. However, while we demonstrate in this study that CC chemokine upregulation occurs in the diaphragm during Pseudomonas lung infection, we have previously reported that inflammatory cells are not recruited to the diaphragm in this model [7], suggesting the existence of alternative functions for chemokines in septic skeletal muscle. 

In this regard, another recently recognized function of CC chemokines is in the regulation of cytokine production. It has been found that both NK cells and CD8+ T cells use RANTES and MIP-1*α* to help drive the Th1 cytokine response [21]. In human monocytes, RANTES is able to directly induce the expression of several proinflammatory cytokines/chemokines (e.g., IL-1*β*, MCP-1) and molecules involved in extracellular matrix digestion (e.g., MMP-9) as well as its own receptor, CCR1 [46]. In the cecal ligation and perforation model of sepsis, it was recently shown that administration of RANTES significantly increases lethality, and this is associated with an increased production of multiple proinflammatory cytokines, which probably underlies the reduction in survival [22]. Taken together, these findings suggest that CC chemokines could act in an autocrine or paracrine fashion to upregulate the expression of other proinflammatory mediators within the diaphragm during sepsis, and thereby contribute to contractile dysfunction.

 There is also evidence for an important contribution of CC chemokines to muscle regeneration and recovery. RANTES is reported to act as a chemotactic factor for myoblasts [47], and gene expression profiling studies have shown an enhanced expression of CC-class chemokines as well as their receptors in injured muscles [48, 49]. Other studies have shown that functional recovery from muscle injury is impaired in mice rendered deficient in MCP-1 or its receptor, CCR2 [17, 18]. Our group has recently shown that several CC chemokines, including MIP-1*α*, are able to stimulate myoblast proliferation [36]. Collectively, these data suggest that at least under certain conditions, chemokines may also play a beneficial role in the repair of diaphragmatic muscle fiber injury triggered by sepsis [5, 6, 24]. The ultimate impact of these CC chemokines upon septic diaphragm function is likely dependent upon achieving the appropriate magnitude as well as spatial and temporal equilibrium of chemokine expression.

## Figures and Tables

**Table 1:** Sepsis-induced increases (fold changes) in prototypical proinflammatory cytokines within the diaphragm.

	LPS administration*		Pseudomonas lung infection^†^
	6 hours	24 hours	48 hours
TNF-*α*	2.3×	1.2×	18×
IL-6	5.8×	1.3×	73×
IL-1*β*	1.6×	1.1×	14.4×

* From [38]. ^† ^From [7].
